# Proteomics-Based Methodologies for the Detection and Quantification of Seafood Allergens

**DOI:** 10.3390/foods9081134

**Published:** 2020-08-18

**Authors:** Mónica Carrera, Manuel Pazos, María Gasset

**Affiliations:** 1 Institute of Marine Research (IIM), Spanish National Research Council (CSIC), 36208 Vigo, Spain; mcarrera@iim.csic.es (M.C.); mpazos@iim.csic.es (M.P.); 2 Institute of Physical Chemistry Rocasolano (IQFR), Spanish National Research Council (CSIC), 28006 Madrid, Spain

**Keywords:** discovery proteomics, targeted proteomics, mass spectrometry, fish allergens, crustacean allergens, mollusk allergens

## Abstract

Seafood is considered one of the main food allergen sources by the European Food Safety Authority (EFSA). It comprises several distinct groups of edible aquatic animals, including fish and shellfish, such as crustacean and mollusks. Recently, the EFSA recognized the high risk of food allergy over the world and established the necessity of developing new methodologies for its control. Consequently, accurate, sensitive, and fast detection methods for seafood allergy control and detection in food products are highly recommended. In this work, we present a comprehensive review of the applications of the proteomics methodologies for the detection and quantification of seafood allergens. For this purpose, two consecutive proteomics strategies (discovery and targeted proteomics) that are applied to the study and control of seafood allergies are reviewed in detail. In addition, future directions and new perspectives are also provided.

## 1. Introduction

Changes in life habits, including food production and manufacturing, have dictated a global increase in adverse food reactions [1,2,3]. Among these reactions, type I IgE-mediated allergies to food components are considered by the World Health Organization (WHO) as the fourth most important public health problem. These food allergies affect an estimated 6–8% of young children and 2–4% of adults with regional variations [3]. The prevalence increase, their life-threatening property, and the costs related to food allergies have dictated the need for improving prevention and treatment strategies [3]. To guarantee consumer safety, several regulations have been implemented (Directive 2007/68/EC), such as the labeling of food allergens that are introduced intentionally [4]. However, some products on the market could contain traces of allergens due to accidental cross-contaminations during the food manufacturing processes.

**Figure 1:**
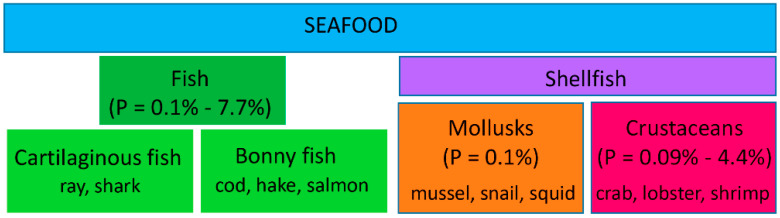
Seafood species classification and their reported allergy prevalence. The classification was performed according to the NCBI taxonomy as described [5]. Examples of each of the species are displayed in the corresponding boxes. The range of the prevalence (P) of each offending food has been taken from References [3,5].

Seafood refers to distinct groups of edible aquatic animals, including crustacean, fish, and mollusk (Figure 1) [5]. Based on culinary reasons, crustacean and mollusk are usually combined as shellfish. The rising consumption of seafood and its derivatives has led to an increase in persistent allergic reactions. The route of exposure is not only restricted to ingestion but includes manual handling and inhalation of cooking vapors in domestic and occupational environments [6].

The diversity of consumed seafood species has challenged the identification and characterization of their allergenic composition for accurate diagnostics and potential therapeutic interventions. Each seafood can contain several allergens, and the allergens causing reactions differ between patients [3,6]. Many allergens are protein families and are very different between these groups. Notwithstanding, β-parvalbumins (β-PRVBs), tropomyosin, and arginine kinase are pan-allergens and induce clinical cross-reactivity [3,6].

To detect the allergens in seafood, tissue aqueous extracts are usually analyzed using Western blot (WB) with the patient sera IgE. This initial approach was then implemented via proteomic analyses, allowing for the identification of proteins after separation by two-dimensional gel electrophoresis (2-DE) and mass spectrometry (MS). The identification and purification of different allergens permitted the generation of both polyclonal and monoclonal antibodies and the development of enzyme-linked immune sorbent assays (ELISA). Notwithstanding, most seafood must be processed before consumption. During processing, proteins can be denatured, modified, and/or hydrolyzed, compromising the immunoreactivity accuracy required by ELISA. New allergenomics techniques, which consider the properties of the allergens, have been developed, allowing for the coupling of identification, detection, and quantification of seafood allergens. MS-based proteomics methods have the additional advantage of being able to target multiple allergens, unlike ELISA, where individual allergens must be detected by different kits. Despite their quality, the dependency on expensive instrumentation and skilled operators are major drawbacks of these approaches. In this review, we summarize the advances in this field.

## 2. Proteomic Workflows: Discovery and Targeted Proteomics

**Figure 2:**
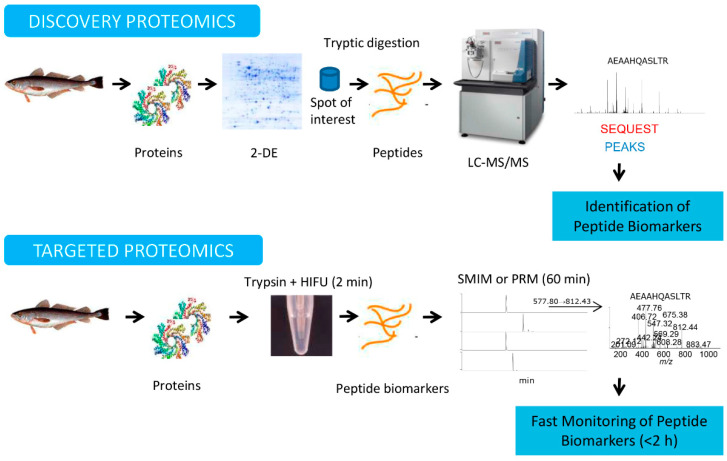
Main proteomics workflows used for the detection and quantification of seafood allergens. Basic workflows used in discovery proteomics and targeted proteomics. In the discovery proteomics approach, a mixture of proteins is separated using two-dimensional gel electrophoresis (2-DE), the spots of interest are in-gel digested with trypsin, the peptides obtained are analyzed using liquid chromatography coupled to tandem mass spectrometry (LC-MS/MS), and the spectra are identified using different database search engines, such as SEQUEST and PEAKS. Specific peptide biomarkers can be selected. In the fast targeted proteomics approach, the mixture of proteins is in-solution digested with trypsin, accelerated using high-intensity focused ultrasound (HIFU), and then the peptide biomarkers selected in the discovery approach can be monitored using selected MS/MS ion monitoring or parallel reaction monitoring (PRM) during mass spectrometry. The monitoring of peptide biomarkers can be performed in less than 2 h.

Figure 2 summarizes the main proteomic workflows used for the detection and quantification of seafood allergens. Two sequential proteomics approaches, namely, discovery proteomics and targeted proteomics, are presented.

Discovery proteomics involves the large-scale analysis of a particular proteome in order to identify protein/peptide biomarkers. By means of a bottom-up proteomics methodology, the protein(s) under investigation are separated to reduce the sample complexity, converted into peptides using enzymes (i.e., trypsin, Asp-N, Glu-C), and the derived peptides are then analyzed using mass spectrometry (MS) [7]. Bottom-up approaches can be organized into two different groups depending on the protein separation step in gel-based or gel-free approaches. In gel-based approaches, 2-DE is the most conventional methodology for the separation of proteins, where proteins are isolated and stained in-gel based on their p*I* and *Mr* [8]. Then, interesting spots can be excised from the gel, digested into peptides with an enzyme, usually with trypsin, and the resulting peptides are analyzed using MS for protein identification. This gel-based method is the most appropriate option for unsequenced organisms, such as some seafood species, in which the identification of proteins is based on the comparison of peptides from related species or via *de novo* MS sequencing [9]. The current availability of specific staining methods makes 2-DE a good approach for the identification of post-translational modifications (PTMs), such as phosphorylations, protein carbonylations, and glycosylations [10,11,12]. With respect to 2-DE image analysis, different programs, such as PDQuest, Progenesis, Melanie, and ImageMaster, are available [13]. In gel-free approaches, also named shotgun proteomics, a mixture of proteins is digested directly with an enzyme (i.e., trypsin) and the complex solution of peptides are separated using liquid chromatography (LC), either alone using reverse phase (RP) columns or combined with multidimensional LC separations as strong anion/cation exchange chromatography (SA/CX)-RP [14]. The eluted peptides are then analyzed and fragmented using tandem mass spectrometry (MS/MS) [15]. By means of protein database search engines, such as Mascot [16], SEQUEST [17], or X!Tandem [18], MS/MS spectra are assigned to potential peptide sequences and then validated using software programs, such as Percolator [19] or PeptideProphet [20]. In the case that the protein is not registered in the protein databases, peptides must be *de novo* MS sequenced [21], either manually or by computer programs, such as PEAKS [22], DeNovoX [23], and Byonic [24]. Notwithstanding, the allergy relevance of the identified proteins using any the previous approaches must be validated using a recombinant produced or purified from natural sources and IgE interaction studies.

Discovery-based quantitative proteomics has been widely used to address the differences in the amount of proteins between different conditions. The most important quantitative proteomics methodologies are isotope tagging via a chemical reaction, such as isobaric tags for relative and absolute quantitation (iTRAQ), tandem mass tag (TMT), and difference gel electrophoresis (DIGE) [25,26,27]; the stable isotope incorporation via enzyme reaction (i.e., ^18^O) [28]; the metabolic stable isotope labeling (such as stable isotope labeling by/with amino acids in cell culture (SILAC)) [29]; and the label-free quantification (i.e., measuring the intensity of the peptides at the MS level) [30]. Shotgun proteomics can also be used to identify and quantify allergens in complex samples using a high-throughput method involving different PTMs as thousands of phosphorylation sites, glycopeptides, and protein acetylations [31,32,33]. The approach known as top-down proteomics analyzes the fragments produced by the dissociation of the intact proteins directly inside the mass spectrometer, avoiding the step of protein digestion [34]. This intact protein analysis is at this time available due to high mass accuracy and the new dissociation mechanisms obtained by the new high-resolution MS (HRMS) instruments [35,36]. Finally, the main goal of discovery proteomics is to compare the resulting peptides and proteins using alignment search tools, such as BLAST, with universal public protein databases in order to select specific peptide biomarkers, which are then utilized in the second phase of the workflow, known as targeted proteomics.

Targeted proteomics is employed to scan the peptide biomarkers selected in the discovery phase with high accuracy, sensitivity, and reproducibility [37]. In this monitoring mode, the MS analyzer is centered on analyzing the peptide(s) of interest using selective/multiple-reaction monitoring (SRM/MRM), mainly on triple-quadrupole (QqQ) mass spectrometers [38]. The monitoring of specific transitions corresponding to appropriate pairs of precursor and fragment ions *m/z* represents a sensitive and selective MS scan mode for detecting and identifying peptide biomarkers [39]. Nevertheless, the implementation of an SRM/MRM study is a time-consuming process, and more importantly, complete MS/MS spectra are not acquired. The MS/MS spectrum of a peptide is extremely important for corroborating its amino acid sequence. Recent procedures, such as SRM-triggered MS/MS in quadrupole-ion trap (Q-IT) mass spectrometers [38], selected MS/MS ion monitoring (SMIM) [40,41], or parallel reaction monitoring (PRM) in an IT or high-resolution Q-Orbitrap (Q-Exactive) instruments [42], are alternative scanning modes that allow for sensitive monitoring of specific compounds, obtaining complete structural information. The sequential windowed acquisition of all theoretical fragment ion spectra (SWATH-MS) [43] is a new advanced targeted data-independent analysis (DIA) mode that is commonly implemented in high-speed acquisition triple-quadrupole-time-of-flight (TripleTOF) mass spectrometers that can identify and quantify large sets of proteins without the prerequisite of specifying a set of proteins prior to acquisition. Targeted-based absolute quantification can be performed using introduced internal standards in the sample with stable-isotope ^13^C- or ^15^N-labeled absolute quantification peptide standards (AQUA) or a concatenamer of standard peptides (QCAT) [44]. Concerning the data analysis, several programs, such as Skyline [45] and SRMCollider [46], are accessible for the analysis of targeted proteomics experiments. The subsequent sections will exhibit the efficacy of these modes for the monitoring of those peptide biomarkers selected in the discovery phase for the detection and quantification of seafood allergens.

## 3. Proteomics Applications for the Detection and Quantification of Fish Allergens

Fish proteins with identified IgE reactivity and registered as official fish allergens by the World Health Organization (WHO)/International Union of Immunological Societies (IUIS) Allergen Nomenclature Sub-Committee [47] are displayed in Table 1.

Among them, β-PRVBs, which are found in high amounts in the sarcoplasmic fraction of the white muscle of fish, are considered as the major fish allergens [48,49]. These proteins have a molecular weight of around 10–12 kDa, an acidic p*I* (3.0–5.0), and three EF-hand motifs (helix-loop-helix), with two of them binding Ca^2+^. The allergenic properties of these proteins are related to their abundance, thermal stability, and resistance to certain gastrointestinal enzymes [3,5,50]. Despite this apparent simplicity, β-PRVBs are indeed a complex family of isoforms differing in their abundance and muscle of expression [5,35]. An advanced discovery proteomics workflow achieved the *de novo* MS sequencing of new β-PRVB isoforms or isoallergens for all the species belonging to the Merlucciidae family [35]. This strategy was performed based on the integration of a common 2-DE bottom-up proteomics methodology with the accurate determination of the *M_r_* of the intact β-PRVBs using Fourier-transform ion-cyclotron resonance (FTICR)-MS and the monitoring of several peptide mass gaps using SMIM. This publication is the report accounting for the higher number of new allergens (25 new β-PRVBs) that were completely *de novo* sequenced by making use of only MS-based techniques. The results allowed for the registration of the sequence of the new β-PRVB isoforms into the UniProtKB [51] and Allergome databases [52] (accession numbers: P86739–P86775). Moreover, the complete sequence of four β-PRVB isoforms (PRVB1 (P86431), PRVB1.1, PRVB2 (P86432), and PRVB2.1 variants) from farmed rainbow trout (*Oncorhynchus mykiss*) were achieved using matrix-assisted laser desorption (MALDI)MS and MS/MS analysis [53]. Such an isoform diversity impacts the stabilization of β-PRVBs as amyloids under gastric-like conditions, and consequently, their resistance to proteases and IgE binding intensity [54,55,56].

In agreement with that, a shotgun proteomics analysis of 15 different fish species, the protein-based bioinformatics analysis and IgE reactive approaches, was used to identify a total of 35 peptides as B-cell epitopes for all the β-PRVBs included in the UniProtKB database [57]

An easy and robust method for fish allergen detection has been developed by utilizing the high-speed, high-resolution, and fragmentation capabilities of the Orbitrap Fusion mass spectrometer implemented with an ultraviolet photodissociation (UVPD) source. Using β-PRVBs as a signature for the allergen detection, the method showed several benefits, such as minimal sample preparation, high sensitivity, high throughput, and a practically complete protein sequence coverage [36].

The rapid detection of β-PRVBs in foodstuffs was developed by our research group using a fast targeted proteomics scanning mode [58]. The strategy is based on the rapid purification of β-PRVBs via treatment with heat (time: 45 min), the acceleration of in-solution protein digestion by high-intensity focused ultrasound (HIFU) (time: 2 min), and the monitoring of several β-PRVB peptide biomarkers using SMIM in a linear ion trap (LIT) mass spectrometer (time: 60 min). The method allows for the rapid detection of the presence of β-PRVBs in any foodstuff, including precooked and processed products, in less than 2 h. Recently, a new method for the quantification of β-PRVBs in food matrices via LC-MRM allowed for the quantification of β-PRVB of flounder (*Paralichthys olivaceus*) at a low level of 0.10 µg/g with an accuracy of <13.3% and a precision of residual standard deviation (RSD) < 18.35% [59].

Regarding other fish allergens, fructose bisphosphate aldolase (39.54 kDa), which is implicated in gluconeogenesis, glycolysis, and the Calvin cycle, is also considered a fish allergen in cod, salmon, and tuna species [60]. This protein was primarily characterized as an allergen using sodium dodecyl sulfate polyacrylamide gel electrophoresis (SDS-PAGE), ELISA, 2-DE, WB, and MALDI-TOF MS in tilapia species (*Oreochromis mossambicus*) [61]. Enolase (isoform β; 47–50 kDa) is an enzyme responsible for the penultimate step of the glycolysis and is also considered a potential fish allergen in cod, salmon, and tuna species [60]. Enolase was characterized as an allergen from the freshwater fish blunt snout bream (*Megalobrama amblycephala*) using 2-DE, WB, and MALDI-TOF MS. Creatine kinase (42 kDa), an essential protein for energetic homeostasis, is considered a potential fish allergen in tuna species that was found using 2-DE, WB, and MALDI-TOF MS [62]. Tropomyosin (33–39 kDa) is a relevant regulator of muscle contraction and is considered a pan-allergen found in shellfish and a potential fish allergen in tilapia and cod species (*Oreochromis mossambicus, Gadus morhua*) [63,64]. The results were performed via immunoblotting and specific IgE ELISA using sera from patients with allergies to tilapia or cod. These four fish allergens (fructose bisphosphate aldolase, β-enolase, creatine kinase, and tropomyosin) are found in the sarcoplasmic fraction of the white muscle of fish and are sensitive to heat treatment and less resistant to food processing as high-pressure treatments compared to the β-PRVBs [65].

Other recognized WHO/IUIS fish allergens include collagen (≈127 kDa) and vitellogenin (≈180 kDa). Collagen was first identified as a fish allergen by Hamada et al. [66] after purification from muscle tissue of tuna and Pacific mackerel skin and demonstrated using IgE reactivity in patients’ sera. Vitellogenin is the major allergen in fish roe (caviar) [67]. The results were obtained using SDS-PAGE, WB using sera from patients, and MALDI-TOF MS analysis.

In addition to these intrinsic allergens, a marked increase over the last ten years has been reported in the prevalence of allergic reactions to fish-borne parasites, mainly to *Anisakis simplex* [68,69,70]. *Anisakis* infects many marine fish species and their storage in industrial freezers for two days or cooking at temperatures above 60 °C kills the parasite but does not destroy the allergens [71]. Fourteen reviewed *Anisakis*-derived allergens (Ani s1-s14) are available in [51,52]. These include proteins, such as paramyosin (100 kDa), tropomyosin (33 kDa), and SXP/RAL-2 family proteins (16 kDa). Like the β-PRVBs, the majority of these *Anisakis*-derived allergens are gastrointestinal-resistant and heat-resistant proteins [68]. A fast targeted proteomics strategy was recently developed by our research group to detect Anisakids in foodstuffs [42]. This technique allows for the rapid direct detection of the main Anisakids species in any foodstuffs in less than 2 h, including processed and precooked products. The analytical methodology is based on the use of a fast purification of thermostable proteins via a heat treatment (time: 45 min), fast trypsin digestion using HIFU (time: 2 min), and monitoring of several Anisakids peptide biomarkers using parallel reaction monitoring (PRM) in a LIT mass spectrometer (time: 60 min). This workflow was also applied for the rapid detection of the allergenic protein Ani s 9, which is characteristic of the Anisakids species. The present strategy allows for the direct identification and detection of Anisakids species in less than 2 h. Currently, this is the fastest method to achieve the direct detection of these allergens independently of the foodstuff encountered.

Label-free, semi-quantitative LC-Orbitrap MS and heavy peptide AQUA LC-QqQ MS methods were used for the quantitation of *Anisakis simplex* proteins in fish [72]. The publication used unique reporter peptides derived from Anisakid hemoglobin and SXP/RAL-2 protein as analytes. Standard curves in a buffer and in a salmon matrix showed limits of detection at 1 μg/mL and 10 μg/mL for MS1 and 0.1 μg/mL and 2 μg/mL for MS2. The proteomic profiling and characterization of differential allergens in the parasites *Anisakis simplex* sensu stricto and *Anisakis pegreffi* were compared using 2-DE, WB, and MALDI-TOF/TOF analysis [73]. Recently, the global proteome profiling of L3 and L4 *Anisakis simplex* development stages and the evaluation of the response of the invasive larvae of *Anisakis simplex* to the ivermectin drug were performed using an advanced quantitative proteomics methodology based on TMT labeling and analysis in a LTQ-Orbitrap Elite mass spectrometer [27,74].

## 4. Proteomics Applications for the Detection and Quantification of Shellfish Allergens

Shellfish allergens include tropomyosin, arginine kinase, sarcoplasmic Ca^2+^-binding protein, myosin light chain 1 and 2, troponin C, and triosephosphate isomerase [47] (Table 1). Among them, tropomyosin has been traditionally considered the main allergen found across the edible parts of either crustaceans (such as shrimp, crab, and lobster) or mollusks (including scallops, oysters, clams, and squid) species [75]. Tropomyosin is a 33 to 38 kDa α-helical protein that forms a coiled-coil structure of two parallel helices containing two sets of seven alternating actin-binding sites. Due to its repetitive coiled-coil structures, tropomyosin retains IgE binding ability even after prolonged heating processing or partial digestion. According to the Allfam database of allergen families [76], 64 allergenic tropomyosins have been identified in animal sources, mainly in shellfish species. The use of discovery proteomics yielded tropomyosin as a major allergen in raw and cooked flower tail shrimp (*Metapenaeus dobsonii*) and in white squid (*Loligo edulis*) [77,78]. The primary structure is highly conserved across various invertebrate species. This seems to be the main reason for high IgE-mediated allergenic cross-reactivity between crustaceans and mollusks, but also other invertebrates, including mites, cockroaches, and parasites [79].

Arginine kinase (38–45 kDa) is also a relevant allergen in shellfish. Although heat labile, arginine kinase has demonstrated IgE binding in heat-treated shrimps, which may be due to the IgE epitopes remaining intact on aggregated arginine kinase [75]. Interestingly, MS has been used to identify arginine kinase as a novel allergen from whiteleg shrimp (*Litopenaeus vannamei*) [79] and crucifix crab (*Charybdis feriatus*) [80]. An early investigation applied proteomics to characterize specific peptides from arginine kinase isoallergens from seven commercial shrimp species [81]. Additionally to arginine kinase, sarcoplasmic calcium-binding protein, myosin heavy chain, hemocyanin, enolase, and glyceraldehyde-3-phosphate dehydrogenase were identified as allergens from banana shrimp (*Fenneropenaeus merguiensis*) muscle [82], and tropomyosin from blue swimming crab (*Portunus pelagicus*) [83]. Similar discovery proteomics approaches have been applied to evaluate the effect of processing on antibody reactivity to allergen variants and fragments of several shellfish allergens. Thus, the sensitizing capacity and allergenicity of arginine kinase after processing was evaluated in crab [84,85]. These investigations showed that enzymatic cross-linking and thermal polymerization of arginine kinase reduces IgE-binding and allergenicity. On the contrary, it was found that heat processing enhanced the overall patient IgE binding to black tiger prawn (*Penaeus monodon*) extracts and increased the recognition of several allergen variants and fragments, such as tropomyosin, myosin light chain, sarcoplasmic Ca^2+^ binding protein, and putative novel allergens, including triose phosphate isomerase, aldolase, and titin [86].

Recently, an innovative methodological approach using in silico bioinformatics identification based on sequence alignment combined with 2D immunoblotting against a serum pool allergic patients and shotgun proteomics confirmed the presence of 24 previously unreported allergens from more than 25,000 proteins of the Pacific oyster (*Crassostrea gigas*) [87]. This investigation demonstrates the presence of multiple novel allergens in shellfish species. Some of these are common to very different allergen sources incorporating animal, including fish and mites, and plant allergens. These results highlight that the comprehensive analysis of unreported allergenic proteins fills a major gap in the current management of patients at high risk of concurrent cross-reactivity to diverse allergen sources.

Targeted proteomics approaches based on LC coupled with high-resolution tandem mass spectrometry (LC-HRMS/MS) has been used to select marker peptides and quantify shellfish allergens, even in the presence of allergens of other multiple sources. An important advantage of this method is its capacity for the multitarget analysis of different allergens in multiple matrixes. Thus, a targeted proteomics method was developed to simultaneously detect and quantify the presence of hidden crustaceans, milk, egg, and soy allergens in fish and swine food products [88]. In this study, tropomyosin was selected as a crustacean allergen marker and PRM was the ion-monitoring technique used. Similarly, the detection and quantification of seven kinds of aquatic product allergens in meat products, including shrimp (*Penaeus vannamei*) and crab species (*Eriocheir* spp., *Scylla serrata*), have been achieved by using a triple quadrupole mass spectrometry (UPLC-QqQ-MS) system [89]. Targeted LC-MS working on MRM and MRM^3^ modes of a hybrid triple quadrupole LIT (linear ion trap) system was used to detect trace contaminations of shrimp and lobster allergens (myosin light chain, myosin heavy chain, arginine kinase, slow muscle myosin S1 heavy chain, fast myosin heavy chain) in salmon lasagna [90]. The procedure set on MRM^3^ mode can detect levels relevant for sensitive allergic individuals with a limit of detection (LOD) as low as 25 µg crustacean per g food. This method targets multiple biomarkers with known sequences for each crustacean food source, thus providing a more comprehensive and reliable approach that is less prone to false-negative or -positive results compared to ELISA systems.

Targeted proteomics has also been exploited in the detection of airborne shellfish allergens in processing plants to prevent occupational asthma and allergenicity. An absolute quantification method and validation of airborne snow crab allergen tropomyosin were first developed by using isotope dilution mass spectrometry [91]. Previously, snow crab tropomyosin (SCTM) was identified as the major aeroallergen in crab plants and a unique signature peptide was identified for this protein. A similar approach was developed to quantify allergenic proteins from northern shrimp in air samples in a processing plant [92]. This procedure indicated the presence of two aerosolized allergens, tropomyosin and arginine kinase, in all areas of the processing plant. These studies show that targeted proteomics is a sensitive and accurate tool for identifying and quantifying aerosolized allergens.

## 5. Proteomics Applications in the Diagnosis of Seafood Allergy

Common to any food allergy, the diagnosis of seafood allergy in clinical practice is based on the identification of the suspected offending food provided by the clinical history followed by its verification using serum-specific IgE determinations, skin prick tests (SPT), and if required, oral food challenges [1,2,3]. Of them, skin testing is relatively noninvasive and provides fast results but its diagnostic reliability highly depends on the extract composition.

Despite the fact that allergic sensitization to fish varies with regions and species and that the worldwide consumption involves about 1000 fish species, the available commercial fish extracts for SPTs only cover 30 species, mostly from the European market. Therefore, the expansion of commercial extracts to a larger number of species is required for covering the worldwide fish allergy diagnosis needs. This limitation also affects shellfish allergy diagnosis reagents.

A recent analysis of 26 fish SPT commercial fish extracts showed a large difference in their total protein concentration (ranging from 0.17–2.94 mg/mL) and high heterogeneity in their protein and allergen composition [93]. Unreported differences in the extract preparation methods ensured the presence of aldolase A and β-enolase but altered the content of β-PRVB, tropomyosin, and collagen allergens. It must be recalled that extracts are usually prepared from raw fish muscles, limiting the presence of collagen that is more abundant in skin and the effects of processing. Furthermore, fish species muscles vary in the number and relative amount of the expressed β-PRVBs, each of which differs in stability and IgE interaction, introducing variables such as isoallergen composition, temperature treatments, and protease content, which is not yet controlled [56]. Indeed, about 30% of the individuals of a patient cohort having cross-reactive anti-β-PRVB IgEs showed oral tolerance to at least one of the fish species tested [94].

The in vivo diagnosis of a shellfish allergy using SPT still requires the use of home-made fresh extracts given the rather low number of commercially available extracts compared to the offending species, as well as their differences in allergen content [95].

## 6. Concluding Remarks and Future Directions

The development and use of proteomic approaches have undoubtedly played a key role in the identification, detection, and quantification of seafood allergens. These advances are becoming essential for the success of the avoidance strategies of allergic consumers and for the improvements in diagnosis reliability. Validated proteomic approaches may soon form part of risk assessment strategies for novel food sources, processing developments, and the standardization of diagnostic reagents. The current allergomics technologies still need further developments.

From the allergen source side and detection actions, all studies have been focused on proteins soluble in aqueous extracts as unique targets, and aggregated and hydrophobic proteins have not been included in the study. An example of such an omission is proteins assembled into amyloid insoluble aggregates that require pretreatment with hexafluroisopropanol (HFIP) for efficient disaggregation of the core [54,55]. A second example is the lipophilic oleosins, which are lost in the conventional extraction protocols [96]. These limitations can be extended to the aerosol form of seafood allergens, which are related to occupational allergies in seafood-transforming plants [6,97]. Then, new methods addressing differences in the solubility of proteins and sampling should be tailored for expanding the detection repertoire. The standardization of extraction protocols and the definition of component-resolve formulas will be essential for the best allergy diagnoses.

From a technical point of view, new mass spectrometer modes for the data-independent acquisition (DIA), such as LC-MS^E^ or SWATH, combined with the high-resolution mass spectrometers (HRMSs), will largely improve the detection and quantification of traces of seafood allergens in different foodstuffs. In addition, DIA coupled with ion mobility mass spectrometry (DIA-IM-MS) will be relevant to investigating the allergen composition in a challenge mixture of ingredient meals. Furthermore, the application of absolute quantitation using AQUA-LC-MRM, the use of capillary electrophoresis (CE) coupled with a top-down proteomics approach to detect intact protein allergens in HRMS instruments, and the employment of new complementary top-down MS/MS fragmentation modes (high-energy collisional dissociation (HCD), electron-transfer-high-collision dissociation (ETDhcD), and ultraviolet photodissociation (UVPD)) for the characterization and *de novo* sequencing of whole allergens are new directions that will provide new valuable insights.

Finally, the knowledge derived from proteomics approaches may allow for the design of protein-based biosensors consisting of a miniaturized device performing real-time in situ analysis. Linking rapid lab-based biosensors with a smartphone readout system will increase the friendly use and accessibility. These devices will make it possible for food industry companies and food control authorities to perform the food routine allergy control test in their own facilities without the need for expensive instrumentations and/or qualified staff. Notwithstanding, technological advances must occur in parallel with their field of application.

## Figures and Tables

**Table 1:** Seafood proteins with known IgE reactivity. Data in the table has been retrieved from the World Health Organization (WHO)/International Union of Immunological Societies (IUIS) Allergen Nomenclature Sub-Committee [47]. Proteins are listed in alphabetical order. X indicates in which seafood groups the protein is described as an allergen, XX indicates a major allergen, and - indicates not yet determined.

Protein	Seafood Source			Function	Molecular Weight (kDa)
	Fish	Crustacean	Mollusk		
Aldehyde phosphate dehydrogenase	X	-	-	Oxidation of aldehydes	41
Aldolase A	X	X	-	Glycolysis	≈40
Arginine kinase	-	X	X	Metabolism	38–45
Collagen	X	-	-	Structural	>100
Creatine kinase	X	-	-	Metabolism	≈40
β-Enolase	X	X	-	Glycolysis	≈50
Glucose 6-phosphate isomerase	X	-	-	Glycolysis	60
Glycealdehyde-3-phosphate dehydrogenase	X	-	-	Glycolysis	≈37
Hemocyanin	-	X	-	O_2_ transport	77
L-lactate dehydrogenase	X	-	-	Metabolism	34
Myosin light chain 1	-	X	-	Structural	17–23
Myosin light chain 2	-	X	-	Structural	17–23
Ovary development-related protein	-	X	-	Unknown	28
Paramyosin	-	-	X	Structural	100
α-Parvalbumin	X	-	-	Ca^2+^-binding	10–13
β-Parvalbumin	XX	-	-	Ca^2+^-binding	10–13
Pyruvate kinase PKM-like	X	-	-	Metabolism	65
Sarcoplasmic Ca^2+^-binding protein	-	X	-	Ca^2+^ buffering	20–24
Triosephosphate isomerase	X	X	X	Glycolysis	28
Tropomyosin	X	XX	X	Structural	33–39
Troponin C	-	X	-	Structural	≈20
Troponin I	-	X	-	Structural	≈30
Vitellogenin	X	X	-	Yolk protein	180
